# Rapid Characterization of Bacterial Electrogenicity Using a Single-Sheet Paper-Based Electrofluidic Array

**DOI:** 10.3389/fbioe.2017.00044

**Published:** 2017-07-26

**Authors:** Yang Gao, Daniel J. Hassett, Seokheun Choi

**Affiliations:** ^1^Department of Electrical and Computer Engineering, Binghamton University, State University of New York, Binghamton, NY, United States; ^2^Department of Molecular Genetics, Biochemistry and Microbiology, University of Cincinnati College of Medicine, Cincinnati, OH, United States

**Keywords:** electromicrobiology, electrogenicity, extracellular electron transfer, exoelectrogens, microbial fuel cells, biosensing arrays, paper-based devices

## Abstract

Electrogenicity, or bacterial electron transfer capacity, is an important application which offers environmentally sustainable advances in the fields of biofuels, wastewater treatment, bioremediation, desalination, and biosensing. Significant boosts in this technology can be achieved with the growth of synthetic biology that manipulates microbial electron transfer pathways, thereby potentially significantly improving their electrogenic potential. There is currently a need for a high-throughput, rapid, and highly sensitive test array to evaluate the electrogenic properties of newly discovered and/or genetically engineered bacterial species. In this work, we report a single-sheet, paper-based electrofluidic (incorporating both electronic and fluidic structure) screening platform for rapid, sensitive, and potentially high-throughput characterization of bacterial electrogenicity. This novel screening array uses (i) a commercially available wax printer for hydrophobic wax patterning on a single sheet of paper and (ii) water-dispersed electrically conducting polymer mixture, poly(3,4-ethylenedioxythiophene):polystyrene sulfonate, for full integration of electronic and fluidic components into the paper substrate. The engineered 3-D, microporous, hydrophilic, and conductive paper structure provides a large surface area for efficient electron transfer. This results in rapid and sensitive power assessment of electrogenic bacteria from a microliter sample volume. We validated the effectiveness of the sensor array using hypothesis-driven genetically modified *Pseudomonas aeruginosa* mutant strains. Within 20 min, we observed that the sensor platform successfully measured the electricity-generating capacities of five isogenic mutants of *P. aeruginosa* while distinguishing their differences from genetically unmodified bacteria.

## Introduction

Electromicrobiology, a field that evaluates the electricity-producing capacity or “electrogenicity” of various bacteria, contributes to novel technologies that address pressing societal issues concerning energy security, environmental protection, bioremediation, and economic development (Rittmann, 2008; Lovley, 2012; Wang et al., 2015). The bidirectional bacterial electron exchange generates environmentally sustainable bioelectricity from organic waste (Logan, 2009, 2010; Babauta et al., 2012). This process can produce value-added chemicals or biofuels, and can perform many other environmentally important functions, such as water desalination, bioremediation, and toxicity detection (Borole et al., 2011; Schröder, 2011; Wang and Ren, 2013; Schröder et al., 2015). These bacterial capabilities have entered a new phase of development with biotechniques in synthetic biology that physiologically and genetically predict and ultimately manipulate bacterial metabolic pathways to improve their electrogenic potential (Rabaey et al., 2009; Alfonta, 2010; Yong et al., 2012, 2014; TerAvest and Ajo-Franklin, 2016). Microbial synthetic biology will aid in the development of fundamentally different strategies to advance electromicrobiology by maximizing the inherent electron-transferring capability of bacteria, translating the technology from the laboratory setting to practical applications. However, there is an urgent need for a sensing technique that characterizes the electrogenic capacity of hundreds of the newly discovered or genetically engineered bacteria. This technique should be adaptable enough to handle the potentially unlimited discovery of such organisms that will likely be made in coming years. Unfortunately, many of the available technologies that could be used are not capable of quickly, simultaneously, and sensitively screening the electrogenicity of bacteria in a low-volume sample.

Recent proposals to use microbial fuel cell (MFC) arrays as a screening tool require a long start-up time (~days), continuous introduction of organic fuels, complex architectural devices, and labor-intensive operation (Biffinger et al., 2009; Cao et al., 2009; Hou et al., 2009, 2011, 2012). This limits the number of distinct sensoring wells on the array only to tens of units, hampering high-throughput quantitative measurements. More specifically, conventional MFC arrays have complex architectures with many tubing ports and fluidic channels operated with external pumps (Hou et al., 2009, 2011, 2012; Mukherjee et al., 2013; Fraiwan et al., 2014). To alleviate these issues, if 96-well MFC arrays were to be constructed to have the conventional MFC configuration requiring individual anolyte and catholyte inlets and outlets, 384 tubes and fluidic accesses would have to be incorporated and operated by several multichannel pumps. Hundreds of electrical wires and contacts for parallel electrical characterization of the individual MFC units may increase the complexity of the device operation, measurement, and maintenance. Furthermore, each MFC unit requires long start-up times for bacterial electrogenic biofilms from their initial bulky planktonic inoculum. Even the latest electrochromic-based colorimetric method that indirectly measures microbial extracellular respiration suffers from low-sensitivity for quantitative measurements. In addition, this technique needs to be further validated as a measure of bacterial electrogenicity because electrochromic color changes based on microbial redox chemical reactions do not necessarily prove microbial extracellular electron transfer (EET) (Yuan et al., 2013; Wen et al., 2014; Zhou et al., 2015). These constraints motivated us to create a new conceptual screening array, such that the rapid and sensitive power assessment can be significantly improved with a compact and simple design, and the development of a much higher throughput array can be easily achieved by simple scalable batch-fabrication.

This work describes a rapid, sensitive, and potentially high-throughput characterization of bacterial electrogenicity from a single drop of culture. Our hypothesis is that through an innovative microscale MFC structure integrated into a single sheet of paper substratum, a simple capillary-driven sensing array can be constructed, resulting in the rapid, sensitive, and high-throughput power assessment of electrogenic bacteria from a microliter sample volume. This hypothesis is based on our extensive experience in biosensors and our recent series of breakthroughs in microfabricating bioelectrochemical systems, all of which suggest that using paper as a device substrate inherently produces favorable conditions for ease, control, rapidity, sensitivity, and parallel analysis of bacterial electrogenicity. Recently, we found that using a paper-based anode/cathode chamber, or reservoir, instead of the usual rigid materials allows for rapid adsorption of bacteria-containing liquid (Fraiwan et al., 2013, 2016; Fraiwan and Choi, 2014, 2016; Choi and Choi, 2015; Choi et al., 2015; Lee and Choi, 2015). This adsorption immediately promotes bacterial cell attachment to the electrode (e.g., biofilm formation), where bacterial respiration can then transfer electrons from the organic substrates to the electrode directly (mediatorless) or indirectly (mediator). MFCs using paper, therefore, require only very short start-up times relative to conventional MFCs as paper substrates substantially reduce the time traditional MFCs require to accumulate and acclimate bacteria on the anode. By exploiting paper as a unique biofilm substratum for bacteria, we introduce a paper-based microbial sensor array as a high-throughput, rapid characterization tool for bacterial electrogenic studies (Fraiwan and Choi, 2014; Choi et al., 2015). For the first time, a 48-well MFC array was fabricated on paper substrates, providing 48 high-throughput measurements and highly comparable performance characteristics in a reliable and reproducible manner (Choi et al., 2015). Within an hour, we successfully determined the electricity generation capacity of two known bacterial exoelectrogens and another metabolically more voracious organism with eight isogenic mutants.

Although the paper-based MFC array platform represents a simple, rapid, and high-throughput (48 wells) screening tool with simple fabrication processes, several major challenges remain. First, the device configuration requires many paper layers to include all the necessary multifunctional components, such as the anode, cathode, and proton exchange membrane (PEM). This demands manual assembly of the device, hampering batch-fabrication for larger-scale array applications and generating artifacts [ultimately leading to relatively large deviation (>2.5%) between MFC units on the array, which is larger than the sensitivity of the device]. In addition, there are potential issues during device fabrication such as misalignment of paper layers and vertical discontinuity between layers, which ultimately decrease power generation and thus sensitivity of the array for bacterial screening. The second challenge was that the MFC array needed to add potassium ferricyanide as an electron acceptor to the cathode. Although this chemical has the advantages of fast cathodic reaction and low overpotential, its use requires more complicated device configurations, fabrications, and operations. Furthermore, the addition of the liquid samples to both the anodic and cathodic reservoirs easily destroys the paper-based proton exchange (or polymer electrolyte) membrane (PEM) and causes an electrical short between the anode and cathode. Third, securing the reservoir to form the fluid and electric connection and connecting to the outside world remains a severe challenge. This is because the effective operation speed and the output sensing performance of the system can be largely limited by these technical bottlenecks. Finally, we could not measure the collective electricity generation harvested from all the bacterial cells placed throughout a paper. Instead, only a small number of exoelectrogens adjacent to the anode contributed to the power generation because the bacterial EET occurs within a short distance from the anode, which impairs the sensitivity of the sensor platform.

**Figure 1:**
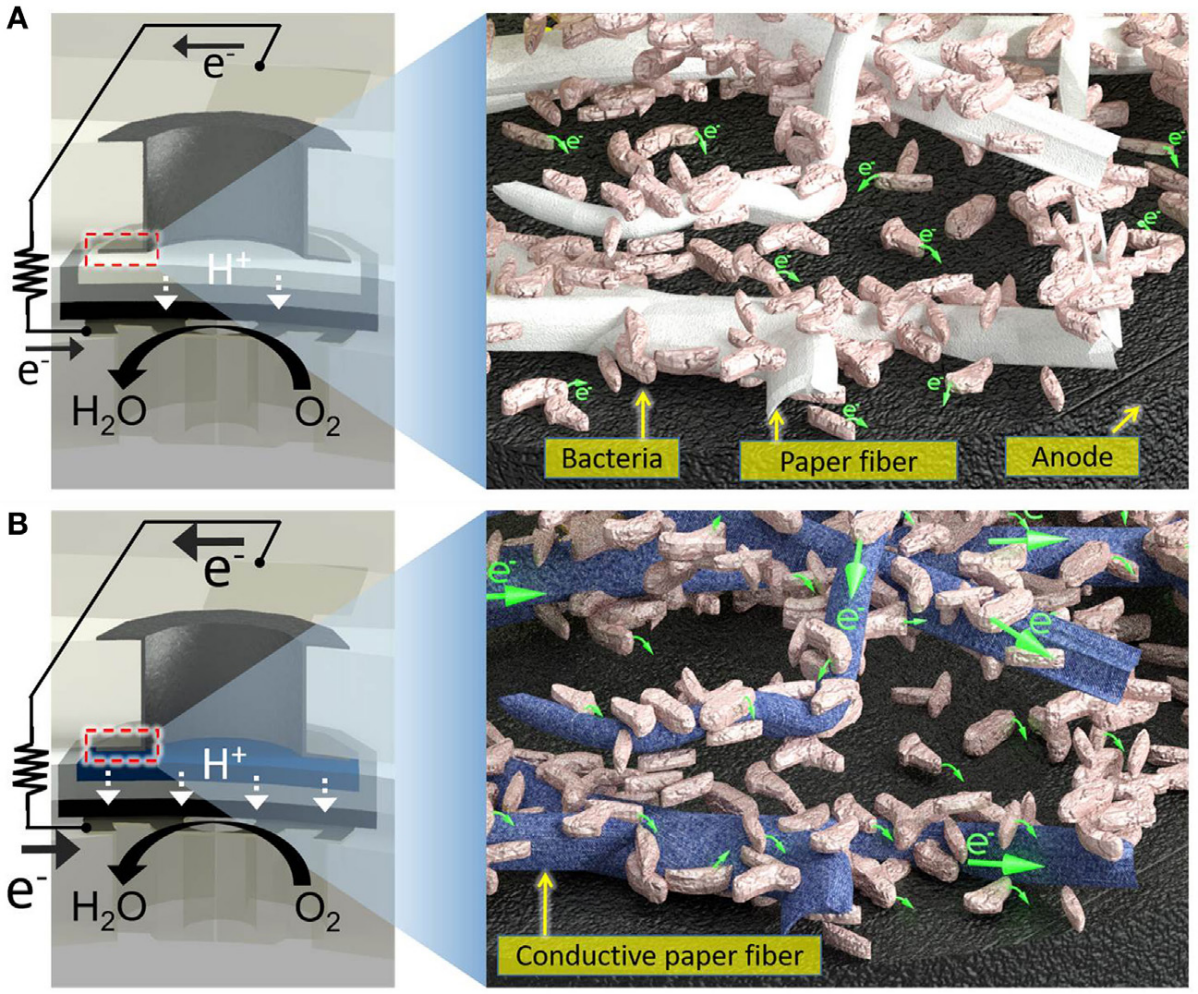
Schematic diagrams of **(A)** a conventional paper-based screening device for exoelectrogens and **(B)** a proposed electrofluidic paper-based device. The conductive reservoir is formed without blocking the pores of paper, which is still hydrophilic. This technique creates a conductive, biocompatible, and porous scaffold for the exoelectrogens placed in each paper reservoir to efficiently transfer electrons to the anode.

In this work, we eliminate the aforementioned major technical hurdles by developing an all-printed, scalable microbial screening array (Figure 1). A three-dimensionally manufactured MFC integrated within a single sheet of paper greatly improves performance, simplifies fabrication and operation, and revolutionizes the scalability of the MFC as an array. Improving the microbial electron exchange with the electrodes in an engineered conductive/hydrophilic paper reservoir and reducing the cathodic bottlenecks by using an air-cathode on paper achieves full integration of a high-performance MFC. Furthermore, the intrinsic capillary force of the paper and the increased capacity from the engineered reservoir allow for rapid adsorption of the bacterial sample and promote immediate microbial cell attachment to the electrode, leading to instant power generation with even a small amount (microliter range) of liquid.

## Materials and Methods

**Figure 2:**
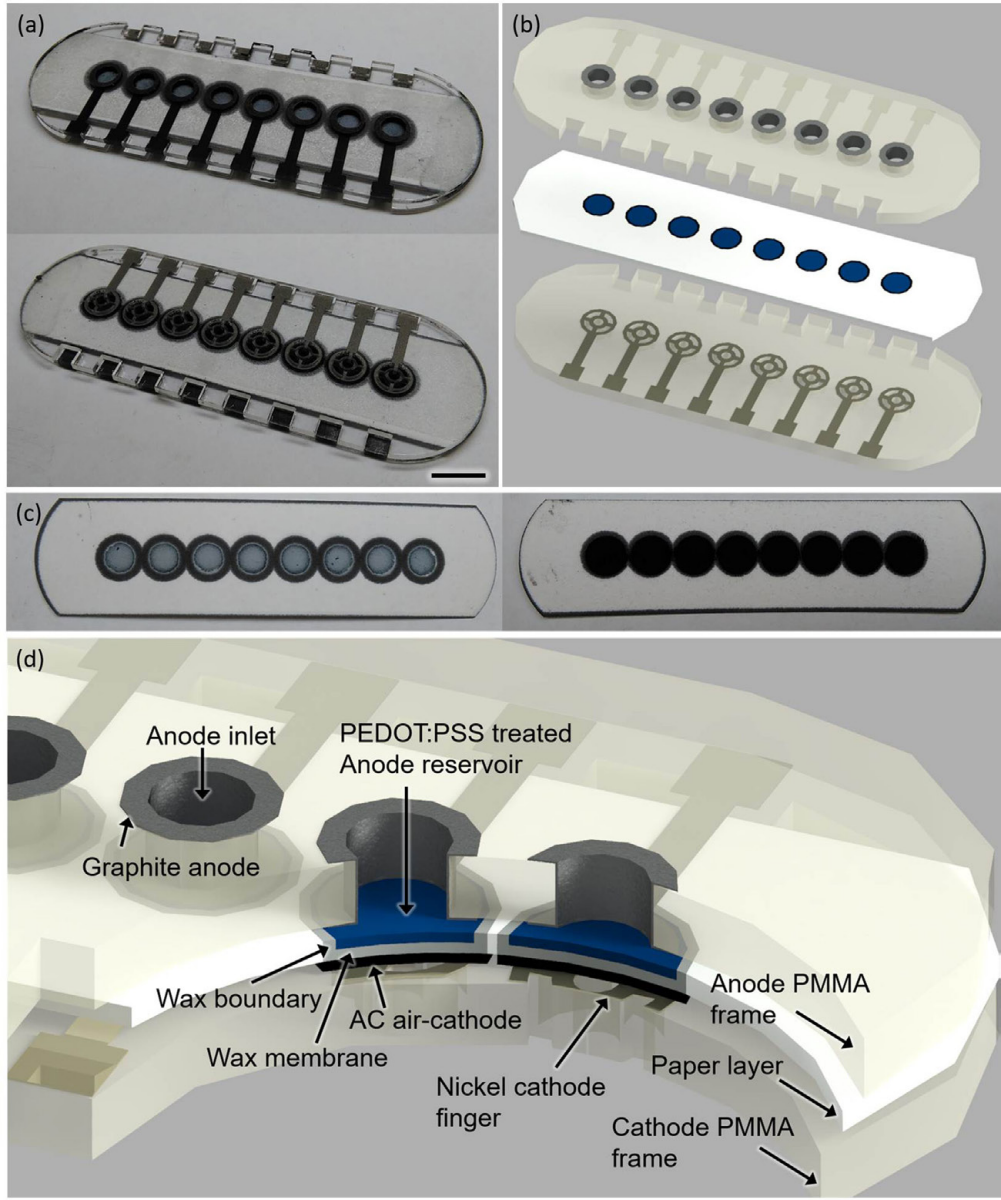
Photo images **(A–C)** and a schematic **(D)** of the sensor platform (scale bar 1 cm). The poly(3,4-ethylenedioxythiophene):polystyrene sulfonate (PEDOT:PSS)-treated anode reservoir [**(C)**, left] and the activated carbon (AC)-based air cathode [**(C)**, right] are fabricated on the opposite side of the paper substrate.

To demonstrate proof of concept of our model system, we built an 8-well single-sheet paper-based screening platform which includes microporous, hydrophilic, and conductive (or electrofluidic) paper chambers, incorporating an air-cathode single-chambered MFC configuration. This potentially revolutionizes the scalability of the device for a high-throughput, cost-effective, large-scale screening technology (Figure 2).

### Reagents

Poly(3,4-ethylenedioxythiophene):polystyrene sulfonate (PEDOT:PSS), Nafion solution, dimethyl sulfoxide (DMSO), phosphate-buffered saline solution (PBS), glutaraldehyde, and 3-glycidoxypropy-trimethoxysilane were purchased from Sigma-Aldrich. Yeast extract and NaCl were procured from Fisher Scientific. Tryptone was purchased from G-Biosciences. Activated carbon (AC) was purchased from Cabot Corporation. A nickel conductive spray for the cathode was purchased from MG Chemicals. Graphite ink was obtained from Ercon, Inc. Filter paper was purchased from the General Electric Company (Whatman 3MM CHR). Poly(methyl methacrylate) (PMMA) sheets were procured from McMaster-Carr.

### Electrofluidic Engineering of Paper Reservoirs

**Figure 3:**
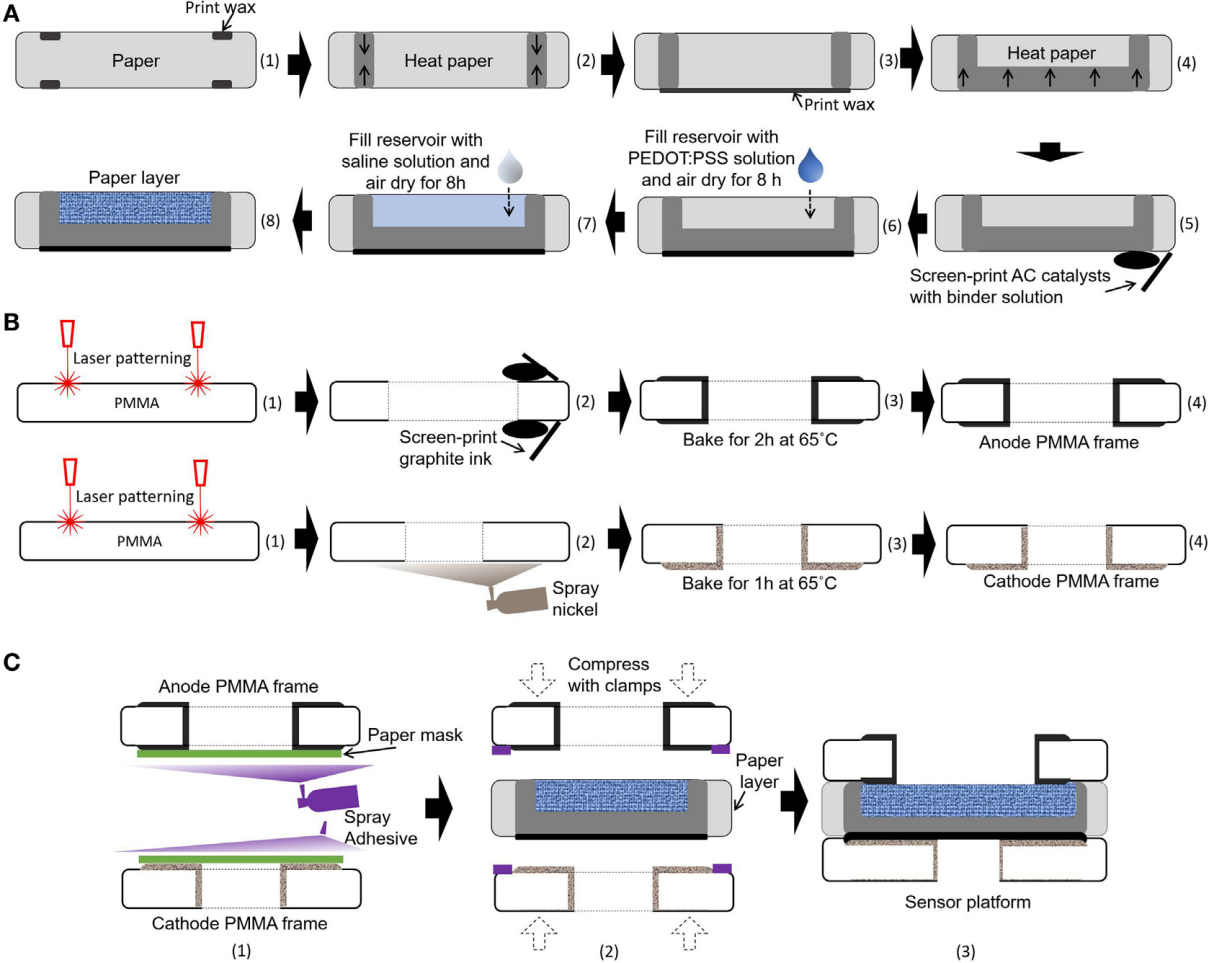
Schematic illustrations of the fabrication process for our sensor platform. **(A)** Integration of microbial fuel cell (MFC) components into a single sheet of paper substrate, **(B)** microfabrication of poly(methyl methacrylate) (PMMA) frames, and **(C)** assembly of the MFC array.

Very recently, the Whiteside group created a novel method that could co-fabricate electronic and microfluidic structures (called “electrofluidic” structures) on paper (Hamedi et al., 2016a,b). They used the water-dispersed conducing polymer mixture PEDOT:PSS as a conductive ink. Upon drying of the ink patterned on paper, the conductive reservoir was formed without blocking the pores of paper, which was still hydrophilic. This technique finds the best-fit solution for our paper-based microbial electrogenic applications by providing a fluidic component that allows mass transfer of ions and an electrical wire that allows conduction of electrons generated from bacterial metabolism. A 20 µl mixture of 1 wt% PEDOT:PSS and 5 wt% DMSO is pipetted into each anode reservoir and air-dried for 8 h (Figure 3A). To further increase anode reservoir hydrophilicity, 20 µl of 2 wt% 3-glycidoxypropy-trimethoxysilane is added to the reservoir and air-dried. This technique creates a conductive, biocompatible and porous scaffold for the exoelectrogens placed in each paper reservoir to efficiently transfer electrons to the anode. Furthermore, this task is the first example of using the electrofluidic engineering technique and revolutionizes the power generation of the MFC.

### Air-Cathode Single-Chambered MFCs on Paper

Air-cathode MFCs offer the best promise for a single-sheet paper-based bacterial screening array, since the cathodic chambers for holding the chemical catholyte can be removed from the system, which reduces device complexity, simplifies fabrication steps, and increases power generation (Lee and Choi, 2015; Fraiwan et al., 2016). Moreover, oxygen is readily accessible, sustainable, and environmentally friendly. In this work, an AC-based air-cathode on paper substrate is constructed with carbon paint/nickel spray that provides structural support and functions as a current collector (Figure 3A). The carbon paint with AC catalysts (15 mg/cm^2^) in a binder solution is screen-printed on a Nickel-sprayed paper zone. The binder solution is prepared by mixing 150 µl of deionized water, 600 µl of isopropanol, and 1200 µl of 5 wt% Nafion solution.

### Fabrication of the Single-Sheet MFC Array

The 8-well MFC sensor array is designed on a single sheet of the filter paper (Figure 2). The hydrophilic reservoir is first defined with hydrophobic wax boundaries on the paper simply by using a commercially available solid-wax Xerox Phaser printer and letting the wax penetrate through the paper with heat treatment (Gao and Choi, 2017). The hydrophobic wax melted into the paper substrate (130°C for 2 min) defines individual MFC units for batch-fabrication and strengthens the paper fiber matrix while retaining its flexibility (Figure 3A). The wax is used as the PEM, as it provides the hydrophobic property of the paper, separating the anodic compartment form the cathode and allowing protons to pass through efficiently. The vertical or horizontal penetration depth of the melted wax is carefully controlled by adjusting the temperature and heating time (130°C for 45 s). The air-cathode is formed on the wax-based PEM. A mixture of PEDOT:PSS and DMSO is pipetted into the defined anodic regions to make it function as an electrofluidic, and 3-glycidoxypropy-trimethoxysilane is added to improve the hydrophilicity. The fabricated single-sheet MFC array is sandwiched between PMMA frames with electrical contacts and is attached with a spray adhesive (3M Super 77, multipurpose adhesive) (Figures 3B,C). Two PMMA frames function as the electrical contacts for anode and cathode and as a supporting structure (Figure 3B). The PMMA frames are micromachined by laser cutting (Universal Laser System VLS 3.5). Then, graphite ink is screen-printed on both sides of PMMA frame for the anode and nickel is sprayed for the cathode. Carbon-based anode material is chosen for the biocompatibility, while nickel is selected for the cathode for high conductivity. After screen printing and spray deposition, the PMMA frames are baked in ventilated oven at 65°C for 2 and 1 h, respectively. A window is made on the cathode PMMA frame to allow the use of freely available oxygen in the air as the electron acceptor.

### Anolyte

To demonstrate the MFC array for studies of bacterial electrogenicity, *Pseudomonas aeruginosa* was selected as our model organism. *P. aeruginosa* has been thoroughly studied with its complete genome sequenced, and there are well-established techniques for their genetic manipulation. For the main microbial EET mechanisms, namely, (a) direct contact, (b) shuttling compound, and (c) conductive type IV pilus nanowires *P. aeruginosa* utilize direct contact electron transfer (mediatorless) and shuttling compounds (mediators) as pyocyanin and pyorubrin (Hernandez and Newman, 2001; Choi, 2015). Our working hypothesis is that the genetic modifications of microbial metabolic/signaling pathways and surface structures greatly affects the electrochemical activity of *P. aeruginosa* strains, and the MFC array provides fast, reliable, accurate information for a quantitative understanding of the resulting electrogenicity. In this work, wild-type PAO1 and five of its strategically engineered mutants *pilT, fliC/pilA, bdlA, lasR/rhlR*, and *pmpR* were tested using the proposed screening platform while deionized water and Luria-Broth (LB) medium are used as the negative controls. The mutants are engineered using classical allelic replacement techniques with sucrose counter-selection (Hoang et al., 2000). Microorganisms are first cultivated in LB medium for 24 h at 37°C, and then centrifuged and re-suspended in fresh LB medium with the cell titers controlled by monitoring the optical density at 600 nm. The detailed information of five genetically engineered *P. aeruginosa* and the rationale behind the genetic modifications for this work is described below in detail.

#### *pilT* (PA0395, Twitching Mobility Protein PilT)

A *pilT* mutant lacks the PilT protein which leads to overproduction of the type IV pili that are always fully extended and incapable of retraction. Previous studies demonstrate that such mutation impairs bacterial twitching mobility, increases the cell adhesion, and promotes biofilm initiation (Whitchurch et al., 1991; Chiang and Burrows, 2003; Burrows, 2012). Denser biofilm and stronger adhesion to glass surface were also observed in a *pilT* mutant when compared to the wild-type PAO1 (Chiang and Burrows, 2003), which could be expected to enhance their electron transfer (Whitchurch et al., 1991; Burrows, 2012), and consequently increase their power/energy efficiency. Studies also suggested the type IV pili may function as antennae by sensing the extracellular environment (Burrows, 2012).

#### *fliC/pilA* (PA1092, Flagellin Type B, PA4525 Type IV Fimbrial Precursor PilA)

The *fliC/pilA* are genes involved in cell motility. A bacteria strain with the mutation on the flagellin structure gene *fliC* does not synthesize the flagellum and, thus, impairs the cells ability to swim toward or away from chemoattractants or chemorepellants, respectively. However, the *fliC* mutant still retains limited swarming capability, indicating that the swarming in *P. aeruginosa* dose not exclusively depend on flagellum but also relates to the function of type IV pili (Kohler et al., 2000). The *pilA* mutation results in the inability to synthesize type IV pili, thus the strains with this mutation are incapable of the twitching motility and limit swarming capability (Farinha et al., 1993; Kohler et al., 2000). Bacteria lacking PilA also affects biofilm formation and leads to a flat and sparse biofilm (Kohler et al., 2000; Klausen et al., 2003). Thus, the *fliC/pilA* mutant is non-motile and impairs biofilm formation with respect to twitching, swarming, and swimming motility.

#### *bdlA* (PA1423, *B*iofilm *D*ispersion *L*ocus)

Regarding *P. aeruginosa* biofilm formation and dispersal, the BdlA protein is a chemotaxis regulator that can facilitate biofilm formation (upregulate) or induce biofilm dispersion resulting in the return to the planktonic form (free-swimming) (Morgan et al., 2006; Li et al., 2014). The dispersion-deficient *bdlA* strain shows decreased dispersion capabilities from biofilm upon both positive (nutrient-induced) or negative (chemorepellent-induced) stimulations (McDougald et al., 2012). The decreased dispersion phenotype could be beneficial to MFC electricity generation as the biofilm formed with *bdlA* strain demonstrates higher resistance and less bacteria reduction toward adverse environmental changes (Petrova and Sauer, 2012; Li et al., 2014).

#### *lasR/rhlR* (PA1430, Transcriptional Regulator LasR, PA3477 Transcriptional Regulator RhlR)

Mutants lacking the LasR and RhlR quorum-sensing (cell–cell communication) regulators forms flat biofilm and leads to overproduction of pyocyanin at the late stationary phase of biofilm formation compared to the wild-type PAO1 (Sakuragi and Kolter, 2007; Dekimpe and Deziel, 2009). Rhamnolipid production is also reduced in the *lasR/rhlR* strain which limits bacteria migration toward nourishment-rich environments (Kohler et al., 2000; Solano et al., 2014).

#### *pmpR* [PA0964, PqsR-Mediated *Pseudomonas Q*uinolone *S*ignal (PQS) Regulator PmpR]

As a part of the global quorum-sensing system, the PQS system is negatively regulated by the product of the *pmpR* gene. Consequently, a *pmpR*-deletion mutant has been shown to possess higher swarming motility and increased pyocyanin production compared to the wild-type PAO1 strain (Liang et al., 2008).

### Measurement Setup

Using an 8-channel pipette, 22 µl of bacterial suspension was simultaneously introduced into each MFC unit in the array. The strong capillary adsorption of the paper promotes bacterial attachment to the anode while the conductive paper reservoirs harvest electrons generated by bacterial respiration. A Scotch Magic™ tape covers the anode reservoir after injecting the sample to prevent evaporation. A data acquisition system (NI, USB-6212) with a customized LabVIEW interface was used to measure the potential between anode and cathode every 1.44 s. To determine the current generated by the MFC units, external resistors (470 kΩ) are individually connected between the anodes and cathodes, and current flows through the resistors are calculated by Ohm’s law.

### Bacterial Fixation and Scanning Electron Microscopy (SEM)

After testing, the MFC array was disassembled and the single-sheet paper layer was gently rinsed with PBS. Immediately after rinsing, the paper layer was immersed in 2% glutaraldehyde solution at 4°C overnight. Dehydration was achieved by submerging the layer in increasing concentrations of ethanol (50, 70, 80, 90, 95, and 100%) for 5 min each. The paper layer was then examined using a FESEM (Supra 55 VP, Zeiss).

## Results

### Paper-Based Electrofluidic Array

**Figure 4:**
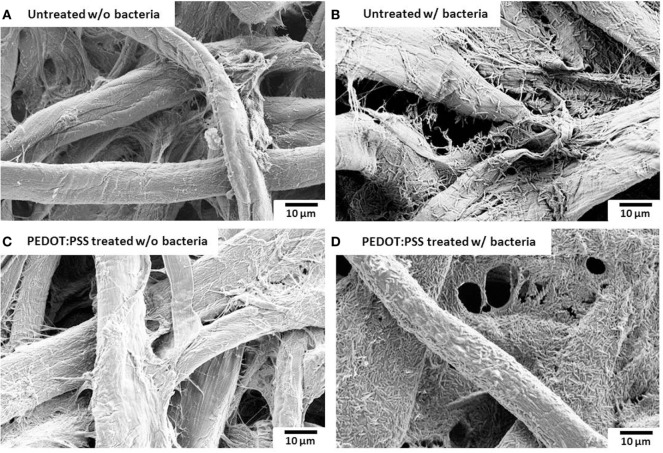
Scanning electron microscopy images. **(A,B)** untreated and **(C,D)** poly(3,4-ethylenedioxythiophene):polystyrene sulfonate (PEDOT:PSS)-treated anode reservoir without bacterial cells or with *Pseudomonas aeruginosa* wild-type PAO1. The scale bar is 10 µm.

**Figure 5:**
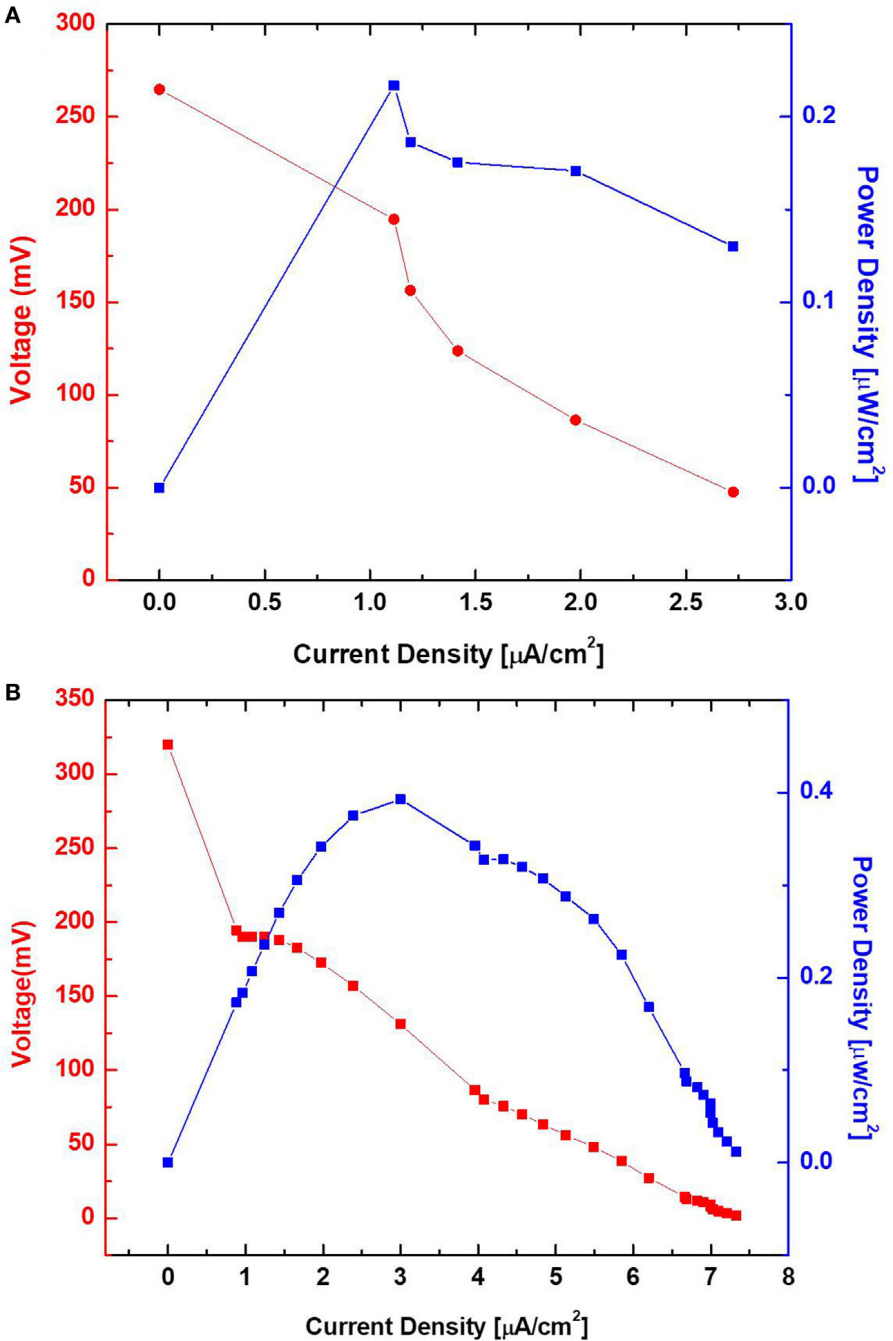
Polarization curve and output power measured as a function of current. Poly(3,4-ethylenedioxythiophene):polystyrene sulfonate-treated **(B)** and untreated **(A)** sensor with wild-type PAO1.

Surface morphology, micro-pores, and the presence of attached bacterial cells of the bare paper and the PEDOT:PSS-treated paper reservoir were assessed by SEM analysis. As shown in Figure 4, the PEDOE:PSS-coated paper shows the same morphology of the bare papers without blocking the paper pores and this macro-porous structure ensures a large surface area and efficient mass transfer to and from the bacteria. The PEDOT:PSS polymer is also very useful as an anode material because of its facile processibility, hydrophilicity, biocompatibility, and stability, features of which lead to fast and dense bacterial adhesion (Jiang et al., 2015). Furthermore, the polymer modification creates more activation centers on anodic surfaces to improve bacterial adhesion and their electrocatalytic activity, increasing electron transfer efficiencies (Guo et al., 2015). Figure 4D shows a higher density of bacteria in the PEDOT:PSS-coated paper reservoir than that on the bare paper alone (Figure 4B), which is well reflected in the polarization curve and output power measured as a function of current (Figure 5). The polarization curve and power outputs are drawn based on the maximum values measured at a given external load. The maximum power density of the PEDOT:PSS-coated paper MFC is about 0.4 µW/cm^2^, which is twice that of the bare paper MFC. Using the polarization curve, we can also estimate internal resistances from the external resistor values where the maximum power densities are obtained. The bare paper anodic reservoir shows the substantially higher internal resistance (800 kΩ) compared to the PEDOT:PSS MFC (200 kΩ). The PEDOT:PSS-coated paper reservoir has a sheet resistance of about 386 Ω/square determined by a four-point probe measurement. The conductivity is enhanced by treatment with the polar solvent, DMSO. Because the conductivity of the paper reservoir is strongly related to the resistance to the microbial electron transfer, the conductive paper modification is helpful for reducing internal resistance, thus ultimately increasing the MFC power performance and providing sensitive power assessment of electrogenic bacteria.

### Open Circuit Voltages (OCVs)

**Figure 6:**
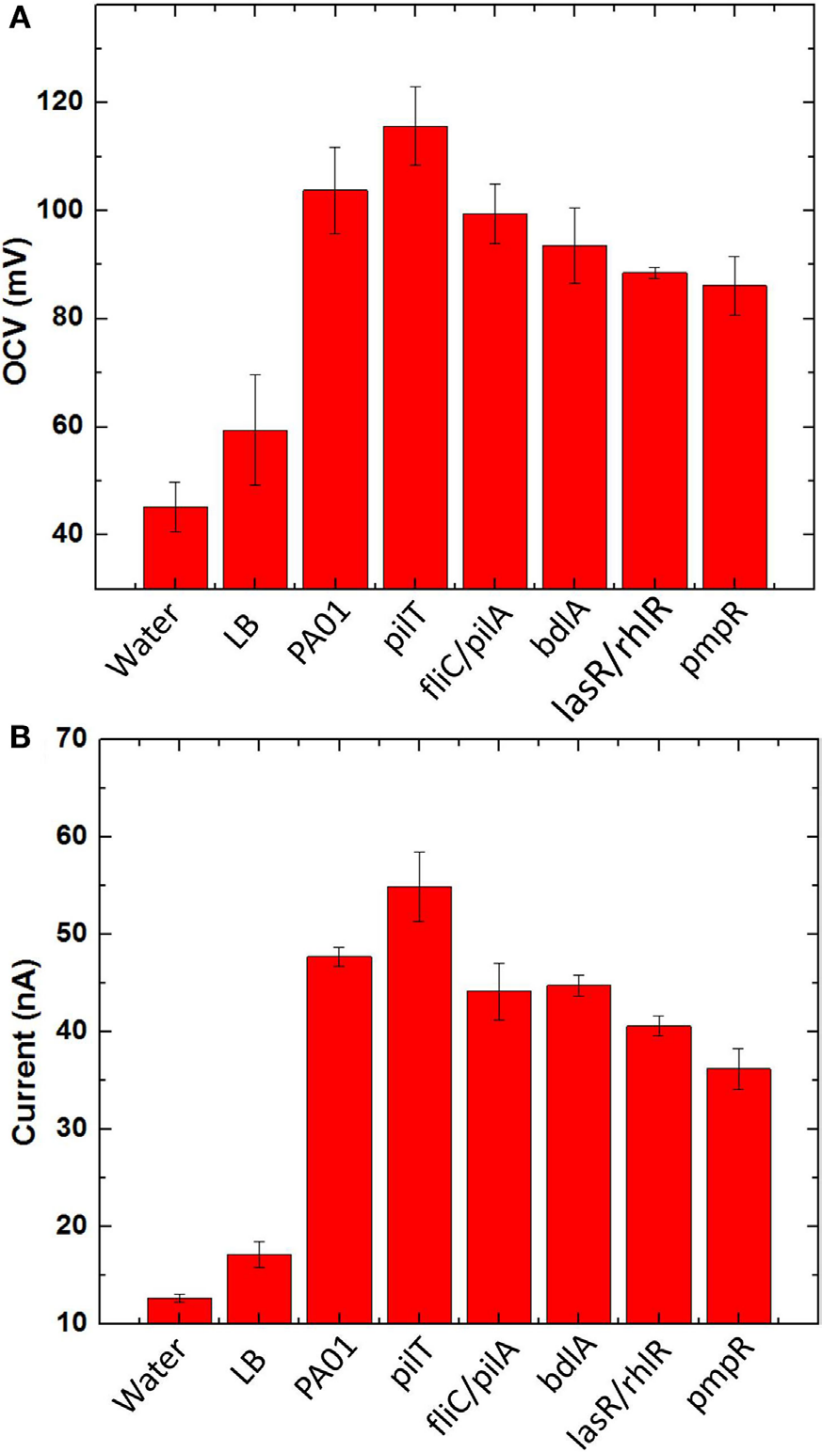
**(A)** Open circuit voltages and **(B)** currents generated from five bacterial species (PAO1, *pilT, fliC/pilA, bdlA, lasR/rhlR*, and *pmpR*) with two negative controls [water and l-broth medium (LB)].

Before we characterized bacterial electrogenicity of the different bacteria strains, we first tested the reliability of the MFC array by measuring the OCV variation from individual device units. The OCVs of the 8-well MFCs generates 1.7% variation with LB medium, which is far less than that of other MFC arrays (25%) and even our previous MFC arrays (2.5%) (Choi et al., 2015). This low percent deviation is mainly because of (i) consistent cathodic reactions from device to device by accessing freely available oxygen in the air and (ii) simplified device configuration, fabrication, and operation by integrating all components in a single sheet of paper. After we confirmed that our MFC array has such a low percentage deviation, we began our experiments with different anodic samples. Eight samples were prepared: deionized water, LB medium, wild-type PAO1, *pilT, fliC/pilA, bdlA, lasR/rhlR*, and *pmpR*. After the anodic samples were introduced into the MFC units and operated under open circuit mode for 15 min, the OCVs from individual MFC units in the array were first compared and the experiments repeated three times (Figure 6A). The OCVs range from 92 to 118 mV with the SD of less than 2%. while the MFC units with water and LB medium have very low OCVs. The highest OCV value is obtained from *pilT* while noticeably second highest is produced from wild-type PAO1.

### Current Generation

To evaluate bacterial electrogenicity, an external resistor (470 kΩ) is used to connect to each MFC unit and the current output through the resistor is calculated 20 min after the load connection (Figure 6B). The negative controls generate a certain amount of current because of ions present in the samples but significantly less current outputs than the other bacterial samples. The current generations revealed significant differences between the respective MFC units. The highest current was observed from the *pilT* mutant, followed by the wild-type PAO1 while the other mutants, *fliC/pilA, bdlA*, and *lasR/rhlR* show the similar electrogenic capabilities but much lower than the *pilT* mutant. The *pmpR* mutant yielded the lowest current.

## Discussion

### Electrofluidic Paper Reservoir for Bacterial Electrogenicity

In the paper reservoir of our previous 48-well MFC for holding the bacterial inoculum, only a few cells adjacent to the anode contributed to the current because the non-conducting paper matrix cannot be an electron acceptor (Figure 1) (Choi et al., 2015). The bacterial EET occurred within a short distance of the anode. Thus, we could not harvest all the electricity that would seem to be available from all the bacteria dispersed throughout a paper reservoir, impairing the device performance, which is inevitable even given the most current technological advances. To overcome this issue, a highly conductive reservoir directly connected to the anode is required. Although electrically conducting papers are available, it is quite challenging to pattern non-conducting papers with conducting materials while enabling them to have open pores and hydrophilic features for subsequent liquid sample introduction. Co-fabricating conducting paths and microfluidic channels structures, namely electrofluidic structures (Hamedi et al., 2016a,b) on paper, creates the best-fit reservoir to efficiently transfer electrons from all the bacteria on the paper of the anode. The application of the electrofluidic technique with PEDOT:PSS over the patterned small paper reservoir of the MFC results in a faster and higher power generation only with microliter sample volume compared to conventional non-conducting paper-based devices. This innovation will be essential in creating a large-scale MFC array for high-throughput and rapid bacterial electrogenic screening.

### Open Circuit Voltages

As an OCV of a device reflects its thermodynamic balance between anode and cathode and all the cathode reactions of the MFC are the same in the array, the OCVs obtained from individual MFCs show the thermodynamic differences of bacterial strains in oxidizing organic substrates. The measured voltages vary significantly between the MFC units, which clearly indicate different thermodynamic reactions of the bacterial strains (Figure 6A). This result indicates that the overproduction of the type IV pili by the *pilT* mutant would enhance bacterial interactions with the anode and reduce the anode overpotential compared to other strains. It should be noted that our previous work with the different bacterial strains had little influence on the OCVs because of their low sensitivity in the non-conductive paper reservoirs (Choi et al., 2015). Although our proposed MFC array provides a reliable screening platform for exoelectrogens with low percentage variation, the OCV values generated from our MFC array are much lower than those of typical MFCs (300–600 mV), which is highly related to overall MFC sensitivity and power assessment. This is mainly due to two fundamental challenges: (i) oxygen diffusion into the paper reservoir is more severe than the conventional MFC device and oxygen abiotically reacts with the anode (Mukherjee et al., 2013) and (ii) an air-cathode configuration suffers from high cathodic overpotential (Xie et al., 2013). Applying a novel oxygen-impermeable membrane for the device and an innovative cathode with high-performance [e.g., solid-state cathodic electron acceptors (Xie et al., 2013)] will be a potential solution to realize a more practical and powerful tool for accurate and parallel analysis of electrogenic bacteria.

### Current Generation

For the hyperpiliated *pilT* mutant, our results demonstrate that hyperpiliation of the type IV pili increases the cell-to-cell, cell-to-surface adhesion and promotes early stage biofilm formation (Giltner et al., 2006), consequently enhancing the bacterial electricity generation (Figure 6B). This is in good agreement with our previous report and other studies (Mukherjee et al., 2013; Choi et al., 2015; Shreeram et al., 2016). The direct EET efficiency of the *pilT* mutant is further boosted by the 3D conductive anode reservoir which provides high surface area for enhanced bacterial attachment and interaction with the anode. The *fliC/pilA* mutant that lacks flagella and type IV pili expectedly generated less current than the *pilT* mutant and wild-type PAO1, which is also supported by previous work (Mukherjee et al., 2013). Despite the hypothesis that it may increase current generation by forming a “less-dispersive” biofilm, the dispersion-deficient *bdlA* strain produces lower current than the wild-type strain. This finding can be explained by the work showing that the *bdlA* mutant shows less enzymatic activity and lacks various proteins such as proteolysis and lipid hydrolysis in contrast to the wild-type PAO1 (Li et al., 2014). Accordingly, the metabolic efficiency of *bdlA* mutant could be reduced in the amino acid/peptide-rich LB medium. In a similar manner, the *lasR/rhlR* mutant also lacks certain proteolytic enzymes, such as LasA and LasB, that are responsible for staphylolysis and elastase activity. As the LasA and LasB proteases are regulated by LasR and partially under RhlR control, the *lasR/rhlR* double mutant fails to degrade milk proteins in an agar medium containing skim milk (Dekimpe and Deziel, 2009). Another factor that could negatively affect MFC performance is the bacteria migration toward nourishment-rich environment. The *bdlA* and *lasR/rhlR* mutants all exhibit limited responses toward nutrient-induced stimulus. Further investigations are required to fully understand the effects of these genetic engineering techniques on MFC current generation.

### Performance Comparison

Table 1 summarizes specifications of prior MFC screening arrays and compares our MFC array to them. Although the electrochromic-based approach provides higher throughput screening capability than the conventional MFC-based method, the technique is limited to specific bacterial species whose EET enables electrochromic color changes. Our recent paper-based MFC screening tools offer several advantages over the conventional technique, including high-throughput and rapid power assessment without using external fluidic pumps or tubes. Also, a low percentage deviation between MFCs can be obtained from simplified and reliable batch-fabrication. This work further improves the potential of the paper-based MFC tools by using air-cathode and electrofluidic technique. However, this platform will not provide general analysis for all type of microorganisms having different electron transfer mechanisms. We need to further study what bacterial electron transfer mechanisms through the paper matrix can be preferably investigated in a more effective way.

**Table 1:** Summary of the characteristics and performances of bacterial electrogenicity screening arrays.

Operation principle	No. of microbial fuel cell (MFC) unites	No. of bacterial species screened	Sample volume per cell (μL)	Fluidic mode reagent drop times	Start time	Deviation between array cells	Reagent/Catholyte	Data acquisition method	External syringe pump	Reference
Electrochromatic	96	12	50	Batch mode/3 times	Instantly	N/A	TMB	Microplatc reader	No	Zhou et al. (2015)
Electrochromatic	96	11	100	Batch mode/3 times	Instantly	N/A	WO_3_	Color scanner	No	Yuan et al. (2013)
MFC based	24	24	600	Batch mode/single time	<2 h	6–14%	Potassium ferri cyanide	Voltage measurement	No	Hou et al. (2009)
MFC based	24	6	400	Batch mode/single time	N/A	N/A	Potassium ferri cyanide	Voltage measurement	Yes/syringe pump	Hou et al. (2012)
MFC based	6	6	1.5	Continuous mode/single time	<2 h	1.4%	Potassium ferri cyanide	Voltage measurement-	Yes/syringe pump	Mukherjee et al. (2013)
MFC based	24	13	600	Batch mode/single time	<20 h	16.4–22%	Air	Voltage measurement	No	Hou et al. (2011)
MFC based (Paper)	48	10	30	Batch mode/single time	20 min	2.5%	Potassium ferri cyanide	Voltage measurement	No	Choi et al. (2015)
MFC-based (paper)	6	6	~20	Batch mode/single time	Instantly	<1%	Potassium ferri cyanide	Voltage measurement	No	Fraiwan and Choi (2014)
MFC based (paper)	8	6	22	Batch mode/single time	Instantly	1.7%	Air	Voltage measurement	No	This work

## Conclusion

We developed a novel paper-based sensing platform for rapid, sensitive, and potentially high-throughput screening and characterization of bacterial electrogenic capacity. This work promotes and accelerates the discovery and characterization of customized and novel exoelectrogens with an in-depth understanding of their electron transfer pathways. With the conductive, microporous, and hydrophilic anode reservoir, the sensitivity of the sensor array is increased by twofold compared to previous studies. Furthermore, with the rapid/strong capillary absorption of the bacteria-containing liquid and bacterial attachment to the conductive/hydrophilic paper fiber matrix, the overall screening/characterization time is dramatically reduced to 20 min. This work provides the first paper-based approach for potentially large-scale biosensing applications, incorporating both electronic and fluidic components, which will augment the emerging field of paper-based electronics or “papertronics.” This work will arguably generate enthusiasm in environment-related research, helping to expand scientist’s understanding of sustainable renewable energy sources that can be scalable, and will inspire the next generation’s scientific minds. Electromicrobiological innovations can revolutionize how bacteria produce energy from a myriad of organic substrates and manage high COD/BOD wastewaters, how they monitor environmental toxicity, how they provide value-added chemicals, and how they drive water desalination with minimum power consumption. In short, such successes will help define a new frontier of potentially transformational research.

## Author Contributions

YG, DH, and SC designed the experiments. YG and SC fabricated devices and conducted the experiments. DH genetically modified *Pseudomonas aeruginosa*. YG, DH, and SC analyzed the data and wrote the manuscript. All the authors discussed the results and revised the manuscript. SC supervised the project.

## Conflict of Interest Statement

The authors declare that the research was conducted in the absence of any commercial or financial relationships that could be construed as a potential conflict of interest.
